# Changes in Resting-State Connectivity following Melody-Based Therapy in a Patient with Aphasia

**DOI:** 10.1155/2018/6214095

**Published:** 2018-03-29

**Authors:** Tali Bitan, Tijana Simic, Cristina Saverino, Cheryl Jones, Joanna Glazer, Brenda Collela, Catherine Wiseman-Hakes, Robin Green, Elizabeth Rochon

**Affiliations:** ^1^University of Haifa, Haifa, Israel; ^2^University of Toronto, Toronto, ON, Canada; ^3^Toronto Rehabilitation Institute, Toronto, ON, Canada; ^4^Canadian Partnership for Stroke Recovery, Heart and Stroke Foundation, Ottawa, ON, Canada

## Abstract

Melody-based treatments for patients with aphasia rely on the notion of preserved musical abilities in the RH, following left hemisphere damage. However, despite evidence for their effectiveness, the role of the RH is still an open question. We measured changes in resting-state functional connectivity following melody-based intervention, to identify lateralization of treatment-related changes. A patient with aphasia due to left frontal and temporal hemorrhages following traumatic brain injuries (TBI) more than three years earlier received 48 sessions of melody-based intervention. Behavioral measures improved and were maintained at the 8-week posttreatment follow-up. Resting-state fMRI data collected before and after treatment showed an increase in connectivity between motor speech control areas (bilateral supplementary motor areas and insulae) and RH language areas (inferior frontal gyrus pars triangularis and pars opercularis). This change, which was specific for the RH, was greater than changes in a baseline interval measured before treatment. No changes in RH connectivity were found in a matched control TBI patient scanned at the same intervals. These results are compatible with a compensatory role for RH language areas following melody-based intervention. They further suggest that this therapy intervenes at the level of the interface between language areas and speech motor control areas necessary for language production.

## 1. Introduction

The goal of the current study was to examine changes in functional connectivity within the right and left hemispheres following melody-based treatment in a patient with aphasia. The long-held notion that recovery from aphasia following LH damage involves compensatory recruitment of the RH [1–4] has been challenged in the last two decades [5]. Many neuroimaging studies showed the effect of treatment-related changes in aphasia predominantly in LH language areas [6–9], and some even suggest that long-lasting RH recruitment is maladaptive for language performance [10–14]. However, melody-based treatments, which have been used for several decades, were developed specifically to harness preserved abilities of the RH in patients with left hemisphere (LH) damage and to facilitate the recovery of speech through RH compensation. Nevertheless, despite evidence for the effectiveness of melody-based therapies in improving speech production in patients with aphasia, the involvement of the RH in this improvement remains an open question [15–19]. While previous neuroimaging studies have examined structural changes and changes in local brain activation following melody-based interventions [20, 21], the current case study focused on changes in functional connectivity within the language network bilaterally. We examined the question whether compensatory processes following melody-based treatment involve changes in connectivity among the right or left hemisphere regions.

### 1.1. Melody-Based Therapy for Aphasia

The use of melody in aphasia treatment is based on the observation that singing and the production of melodic speech are often intact, even when standard speech is impaired in patients with nonfluent aphasia [22–24]. In Melodic Intonation Therapy (MIT), the most commonly used melody-based therapy, participants repeat phrases with a simplified and exaggerated prosody, characterized by a melodic component of two notes and a rhythmic component of two durations [16, 25]. The protocol of MIT also includes tapping the rhythm with the left hand while repeating the melodic phrases [18]. Other melody-based therapies modify the melodic structure of the phrases or expand some of the musical elements [16, 26]. MIT has been recommended for use with nonfluent aphasia patients with poorly articulated speech, severe disorders in repetition, and relatively good auditory comprehension [19]. MIT has been employed primarily with patients with aphasia due to stroke; however, MIT or similar therapies have also been administered to patients with other neurologically based speech and language disorders, such as traumatic brain injury (TBI, e.g., [26]). Two review papers of MIT and other melody-based therapies [16, 19], which together include over 600 patients, concluded that there is positive evidence for the benefit of these therapy methods (although many studies did not measure generalization to spontaneous communication). The efficacy of these treatment methods was evident in both subacute [27] and chronic [16] patients. Nevertheless, a recent randomized control trial, with 17 chronic patients that received MIT, did not find stable maintenance of the effects at 6-week follow-up and no generalization to untreated items [28].

### 1.2. Mechanism and Lateralization

Despite the common use of melody-based therapies, and the positive evidence for their effectiveness, the underlying mechanisms and the role of the melodic component in language recovery are still unresolved [16, 19]. Other critical ingredients may be the rhythmic cueing provided during treatment [29] or the slow articulation of connected syllables which enhances auditory-motor feedback and increase inner rehearsal [23, 25]. Processing of music and prosody, and spectral processing more generally, have traditionally been associated with the RH [30–33]. RH regions have also been associated with the production of sung versus spoken output [34–37]. The preserved ability of patients with aphasia to sing, despite difficulty in producing spoken output, underlies the assumption that melody-based treatments should recruit RH regions [22].

The current evidence, from functional imaging studies, for the involvement of the RH in melody-based treatment is mixed. An early PET study ([38], *N* = 7) and several smaller fMRI studies ([39–41]; total *N* (across studies) = 5) showed an increase in LH activation and decrease in RH activation in patients who benefitted from melody-based treatments. However, some interpreted such a decrease in RH activation as reflecting greater efficiency of the RH language processing [42], and others ([17, 18]; total *N* = 4) showed bilateral increases in activation with a more prominent increase in right frontal activation. Recently, a case series with patients with aphasia showed increases in right lateralization in four out of five subacute patients (i.e., within three months poststroke) following MIT but no right lateralization in chronic patients (i.e., greater than 1 year postonset; [43]).

Structural-imaging studies show more consistent evidence for the involvement of the RH in melody-based therapy. 11 patients undergoing MIT showed an increase in RH white matter volume and a correlation of behavioral improvement with changes in the right IFG pars opercularis [44]. Diffusion tensor imaging (DTI) measures show an increase in the number of fibers in the right arcuate fasciculus in 7 patients undergoing MIT [20, 21] and showed a marginally significant correlation with improvement [20]. In addition to the effects of melody, this can be the result of the left hand tapping that accompanies treatment [20], as was also shown in naming treatment studies that do not involve melody [45, 46].

Finally, two transcranial brain stimulation studies showed that excitatory stimulation of the right posterior IFG has improved the effects of MIT in some of the participants [15, 47]. It should be noted that although these studies show a role for right frontal areas in treatment improvement, they do not compare it to the role played by the LH.

### 1.3. The Current Study

The current study aims to examine the underlying brain mechanisms associated with melody-based treatment by looking at changes in the functional connectivity in the language network. In contrast to the ambiguous interpretation of local activation changes (in which a decrease in activation in the RH may reflect less reliance on the RH or more efficient processing in the RH), functional connectivity with RH regions is more clearly associated with increased involvement of the RH. We examined the changes in resting-state connectivity in the language network of a patient with chronic nonfluent aphasia associated with melody-based treatment. Resting-state measures do not depend on the patient's level of language performance, while still depicting connectivity in the language network [48–52]. Treatment-related changes in resting-state connectivity have been shown following motor recovery in motor areas [53] and following aphasia treatment in both the default mode [54] and language [55] networks. Patient connectivity analyses were compared to a control patient who did not receive therapy during the same time interval. We predicted that melody-based treatment would enhance the connectivity among right frontal language areas (i.e., language production homologues), as well as between these areas and motor regions associated with planning and execution of speech. We did not expect to find such enhancement of connectivity among LH language areas in our patient or in the untreated control patient.

## 2. Methods

### 2.1. Participants

**Figure 1:**
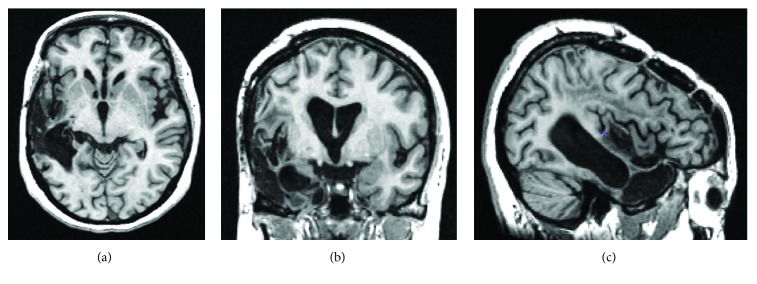
MR T1-weighted image of the patient's brain in (a) axial, (b) coronal, and (c) sagittal views. The patient presents with an extensive area of encephalomalacia within the left cerebral hemisphere involving the temporal and frontoparietal lobes with volume loss.

JV was a 48-year-old right-handed female at the time of injury, with 16 years of education. She is a native speaker of Tagalog, and was a fluent speaker of English as a second language. She sustained a moderate-severe traumatic brain injury (TBI) secondary to a fall from a ladder onto a concrete floor. A CT scan performed on the day of the injury indicated left frontal and temporal subdural hematomas (Glasgow Coma Scale (GCS) score not available), which were evacuated in an urgent craniotomy. JV was in a coma for five to six weeks. Unfortunately, she sustained a second TBI three months later, as a result of another fall while in hospital, at which time her language symptoms worsened. MRI at the time of the second TBI showed evidence of a new subarachnoid hemorrhage in the left medial temporal sulcus, in addition to underlying encephalomalacia in the left frontal and left temporal lobes associated with the initial hematomas (see Figure 1). The results of neuropsychological assessment conducted 25 months after the second injury and language assessment conducted 36 months after the second injury (immediately before therapy) show moderate-severe nonfluent aphasia, characterized by limited spontaneous speech output, stereotypical utterances, moderate comprehension deficits, and good use of gestures and facial expressions to support communication. The patient also presented with moderate apraxia of speech (based on tasks for assessing apraxia of speech [56]), characterized by audible groping, sound distortions and substitutions, articulatory self-corrections, difficulty with words of increasing length, delayed response initiation, and slow rate of speech. She had normal visuoperceptual and hearing abilities. She appeared to respond well to musical stimulation, including melodic and rhythmic cueing, during informal diagnostic testing. Detailed language and neuropsychological assessment findings are presented in Tables 1 and 2.

The control TBI patient, GB, was a right-handed female, native speaker of English, with 13 years of education who was 54 years of age at the time of injury. She had sustained a moderate to severe TBI secondary to a motor vehicle accident. Her GCS at the scene was 5 and declined to 3 at the time of admission to the emergency department. A CT scan performed on the day of injury indicated a left temporal subarachnoid hemorrhage as well as an intraventricular hemorrhage. GB was in a coma for approximately 1 week following the accident. Neuropsychological assessment at 28 months postinjury did not indicate any persistent language deficits. Although the control patient is a native English speaker, while the treated patient speaks English as a second language, we do not expect this to affect the results because we are not comparing between them on language performance or on a language task-related functional imaging. Ethics approval was granted for the study, and both participants had signed an informed consent.

### 2.2. Treatment Description

Thirty-six months after her second injury, as part of the current study, JV started receiving a melody-based treatment, which was a modified version of MIT [25, 28]. Therapy was developed jointly by a speech language pathologist and music therapist and administered in English, remotely via Skype® by the accredited music therapist (coauthor Cheryl Jones), specialized in Neurologic Music Therapy [60]. All treatment stimuli were trained using the musical elements of melody and rhythm. A unique melody, distinct in shape and rhythmic pattern, was assigned to each target phrase and served as a timing template to support word retrieval and the fluency of word production. This is in contrast to the standard MIT protocol, which uses only two to three tones for all phrases. Rhythmic cues served to support oral motor timing impairments and word fluency [60].

Each target phrase, and its associated unique melody, was presented following the standard MIT hierarchy (e.g., [25]): the clinician, accompanied by an electric keyboard, first played and hummed the melody and then sang the target words. The participant then sung the phrase in unison with the therapist for a number of repetitions, with the therapist gradually omitting words until the participant was singing independently. These steps were repeated until the patient could sing the target phrase without support. The participant's son or daughter, present at every session, tapped her left hand, as per the MIT protocol.

Melody served as the primary cue to support word production within the target phrase, and the unique melody was played up to five times, as required, allowing the participant to gradually fill in the melody with the target words. Initially, the participant required maximal melodic cueing (i.e., 5 cues), but this need decreased (i.e., to 1-2 cues) over the course of treatment. Rhythm served as the secondary cue, whereby the rhythm of the target phrase was tapped on her left arm in order to help the participant overcome hesitations or sustained pauses during a phrase. Additional cues included modelling of oral-motor placement and providing the first word in the target phrase. The need for these cues, however, significantly reduced as treatment progressed. In addition to the hierarchical progression of cues, the treatment was designed to enhance generalization, by gradually decreasing the structure of the target phrases, thereby increasing the linguistic difficulty. Namely, the treatment involved progressing through 5 steps: steps 1 to 3 primarily involved repetition; step 4, sentence completion; and step 5, phrase production (in response to target questions). Novel responses were encouraged in steps 4 and 5.

The primary goal of steps 1 to 3 was to use melodic and rhythmic cues to encourage word retrieval and fluency in sentences of increasing length and complexity. The participant was required to produce target phrases with and without melodic intonation. The primary goal of step 4 (sentence completion task) was to encourage generalization beyond trained phrases, by asking the participant to generate novel words at the end of each rehearsed target phrase. In step 5, the primary goal of treatment was for JV to produce fully self-generated phrases, using two tones to produce nonrehearsed responses to target questions. Untreated items were also created and matched to all treatment stimuli in terms of number of syllables, syntactic complexity, and relevance to the participant's life and interests. These items were not treated and served for testing before, immediately after, and eight weeks following treatment (see outcome measures).

### 2.3. Treatment Stimuli

Treatment stimuli were created based on an “interest” inventory completed by JV and her family, whereby the participant listed her primary hobbies and interests, important members of her family and friend groups, details from her past, and her speech-related goals. Treatment stimuli were phrases on a continuum of difficulty in terms of syllable length and syntactic complexity. Shorter, simpler (e.g., imperative and wh-question) phrases were used initially, followed by longer, declarative present- and past-tense phrases (based on HELPSS hierarchy, [61]). The target phrases were categorized into five distinct treatment steps: (1) 2–4 syllables (e.g., “How are you?”); (2) 5–7 syllables (e.g., “Please do the dishes”); (3) 8–10 syllables (e.g., “I drink coffee every morning”); (4) sentence completion cues (e.g., “For dinner I will make…”); and (5) question probes (e.g., “What did you do yesterday?”). There were seven target phrases in steps 1 to 3, nine target phrases in step 4, and 10 target questions in step 5.

### 2.4. Treatment Schedule/Protocol

JV started receiving treatment 36 months after her second injury and received treatment three days a week for 16 weeks, for a total of 48 sessions. Each session lasted approximately 30 minutes. At the beginning of each session, three previously learned phrases were rehearsed, to encourage maintenance and continued practice of all treated items. Progression through steps 1 to 5 occurred if either (a) the participant produced 80% of the target phrases with 80% accuracy on two consecutive sessions or (b) the participant completed 9 sessions of therapy within a single step. Target accuracy was measured as the proportion of syllables correctly produced within the target phrase (for steps 1 to 3) and the appropriateness of the responses given to sentence completion and question probes (for steps 4 and 5). Each melody and phrase was notated and emailed to the participant, who was instructed to practice the target phrases between sessions for 30 minutes, three times per week. The participant was also required to sing personally significant songs for ten minutes a day, five days a week, in an attempt to reinforce word retrieval through familiar melody and word associations. These activities were monitored by the patient using a “homework log.” These logs indicated very good compliance of the patient with the homework.

### 2.5. Primary Outcome Measures

Both treated and untreated items were administered pretherapy, immediately posttherapy, and at eight weeks posttherapy by a registered speech-language pathologist who was not involved in the treatment (coauthor Tijana Simic). The primary outcome measure for steps 1 to 3 was the ability to repeat target phrases accurately (measured as the *proportion* of correctly produced syllables in each phrase). In steps 4 and 5, the primary outcome measures were the *raw number* of syllables produced in appropriate responses to sentence completion cues (step 4) and questions (step 5). Syllables produced in inappropriate responses (e.g., that did not fit the context of the cue or question probe) were discarded and not included in the raw counts. All outcome measures were taken using spoken, not melodic, cues.

### 2.6. MR Image Acquisition Protocol

Whole head MR scans were acquired on a General Electric (GE) Signa-Echospeed 1.5 Tesla high-definition scanner, located at Toronto General Hospital—University Health Network, using an eight-channel head coil. The high-resolution 1 mm isotropic T1-weighted, three-dimensional radio-frequency spoiled-gradient recalled-echo (SPGR) images were acquired in the axial plane utilizing a 25 cm field of view (FOV) (TR/TE/TI = 12/5/300 ms), flip angle = 20°, slice thickness = 1 mm no gap, 160 slices, and matrix 256 × 256. Resting-state BOLD fMRI data were acquired with the following imaging parameters: TR/TE = 2000/40 ms, flip angle = 85°, FOV = 22, slice thickness 5 mm with no gap, 32 slices with 4800 images, and matrix 64 × 64. Resting-state data were collected at three time points. For JV, this was at 25 months, 35 months, and 39 months after the second injury, with treatment occurring between 36 and 39 months postinjury. Resting-state data for the control patient GB was collected at 28 months, 32 months, and 36 months postinjury, with no speech and language treatment. Additional sequences include DTI and axial fast spin-echo PD/T2-weighted images, which are not presented here. All sequences were obtained with a 22 cm FOV. The entire scanning session lasted approximately 55 min.

### 2.7. Resting-State Connectivity Analysis

#### 2.7.1. Preprocessing

The preprocessing of resting-state data was performed using SPM12 (Wellcome Department of Imaging Neuroscience, London, UK; http://www.fil.ion.ucl.ac.uk/spm). The preprocessing steps included removal of the first five scans, slice-timing correction, rigid-body motion correction and unwarping, spatial normalization to the Montreal Neurological Institute (MNI) space, and smoothing with an 8 mm FWHM Gaussian kernel.

#### 2.7.2. Functional Connectivity

Functional connectivity analysis was performed using a region of interest (ROI) to ROI approach within CONN's functional connectivity toolbox (Whitfield-Gabrieli and Nieto-Castanon 2012; http://www.nitrc.org/projects/conn). We selected anatomical ROIs in bilateral frontal areas: inferior frontal gyrus (IFG) pars opercularis (Operc), pars triangularis (Tri), and pars orbitalis (Orb); precentral gyrus (PreC), insula, and supplementary motor area (SMA). Subregions within left IFG (opercularis, triangularis, and orbitalis), known to be involved in language production [62], were previously used as seed regions for the language network in resting-state studies [48, 51, 52]. Their homologous regions in the right IFG were shown to be involved in melody-based treatments, in structural-imaging and brain stimulation studies [15, 44, 47]. The left precentral gyrus, insula, and SMA are typically involved in motor sequence planning for articulation and initiation of speech [63–65], and the right SMA was also shown to be involved in melody-based treatments [21]. A mask was created to encompass these ROIs using the automated anatomical labeling (AAL) atlas [66].

Sources of physiological noise (based on white matter and cerebrospinal fluid segmentation) and movement covariates (motion correction and scrubbing using Artifact Detection Tools; ART http://www.nitrc.org/projects/artifact_detect) were regressed out of the data. Data was also bandpass filtered to retain low-level frequencies (0.009 < *f* < 0.08 Hz). Semipartial correlations were then computed using the residual data across all ROIs, for each time point (T1-baseline, T2-pretreatment, and T3-posttreatment) and participant (treated versus control patient). In semipartial correlations, the effects of all other regions on the *target* region alone are held constant while computing the correlation between the source and the target region. Therefore, semipartial correlation between two regions is not symmetric and depends on which region is the target. The correlations were then transformed into *z*-scores using Fisher's *r* to *z* transformation.

Similar to the approach taken by Sandberg et al. [67], difference matrices were computed to determine the level of increase and/or decrease in functional connectivity across time points. A difference matrix was calculated by subtracting post- minus pretreatment functional connectivity to identify changes during the “treatment period” (or its equivalent in the control patient; T3 − T2) and by subtracting pretreatment minus baseline functional connectivity to identify changes during the “baseline period” (T2 − T1). Significant differences in correlational changes across time points (treatment versus baseline period; separately for each participant; *p* < 0.05 (FDR corrected for 66 comparisons)) were determined using Fisher's test.

## 3. Results

### 3.1. Treatment Outcomes/Behavioral Results

The proportions of correctly produced syllables before and immediately posttreatment, for both treated and untreated phrases, are presented in Table 3(a) for the repetition task (steps 1 to 3, collapsed). The *number* of syllables produced in appropriate responses to sentence completion and question probes (steps 4 and 5, resp.) are presented in Tables 3(b) and 3(c), respectively. The Wilcoxon signed-rank test for the comparison of two related samples showed no significant difference in pretreatment performance between the treated and untreated phrases for the repetition (*Z* = −0.967, *p* = 0.333), sentence completion (*Z* = 0.853, *p* = 0.394), and probe question (*Z* = −0.272, *p* = 0.785) tasks. Given the varying task demands, analyses for these three tasks were completed separately.

The Wilcoxon signed-rank test for related samples was used to assess the treatment effect on syllable production when repeating sentences of increasing length and complexity (steps 1 to 3) and compared to performance on untreated stimuli sets. Three comparisons were made with these data; using the Bonferroni correction, alpha was set at 0.017. JV's ability to repeat syllables within treated phrases significantly improved pre- to posttreatment (*Z* = 3.061, *p* = 0.002) and was significantly better than her ability to repeat syllables in matched untreated phrases posttreatment (*Z* = −3.313, *p* = 0.001); this treatment effect was maintained at eight-week follow-up (*Z* = 2.552, *p* = 0.011).

In the sentence completion task (step 4), five comparisons were made using the Wilcoxon signed-rank test for related samples; thus, alpha was set at 0.01 (Bonferroni correction). The difference between pre- and posttreatment tests in the number of syllables produced by JV when given a sentence completion cue approached, but did not reach, significance (*Z* = 2.319, *p* = 0.020); no significant increase was seen from pretreatment to the eight-week follow-up either (*Z* = 1.582, *p* = 0.114). Visual inspection of the data for untreated phrases suggested a generalization effect to untreated items in this task. Indeed, when comparing treated and untreated items posttreatment, no significant difference was found (*Z* = 0.704, *p* = 0.481), indicating overall improvement of both treated and untreated items over time. We therefore also compared improvement in untreated items pre- to posttreatment (*Z* = 1.975, *p* = 0.048) and pre- to eight-week follow-up (*Z* = 2.232, *p* = 0.026). These comparisons approached, but did not reach, significance.

Finally, JV's ability to answer questions (step 5) was also assessed using the related-sample Wilcoxon signed-rank test; five comparisons were again made here; thus, alpha was set at 0.01. JV's ability to answer treated questions significantly improved pre- to posttreatment (*Z* = 2.814, *p* = 0.005), and this was maintained at eight-week follow-up (*Z* = 2.692, *p* = 0.007). In addition, her ability to answer trained questions compared to untreated questions posttreatment was significantly better (*Z* = −2.561, *p* = 0.010). As in the sentence completion task (step 4), we assessed whether generalization to untreated items occurred but did not find significant improvements in performance on untreated stimuli pre- to posttreatment (*Z* = −1.826, *p* = 0.068) and pretreatment to eight-week follow-up (*Z* = 1.841, *p* = 0.066).

JV was reassessed on various language measures following treatment (Table 1) but, apart from better performance on the BDAE responsive naming task, did not show notable improvements on these tasks.

### 3.2. Resting-State Connectivity

**Figure 2:**
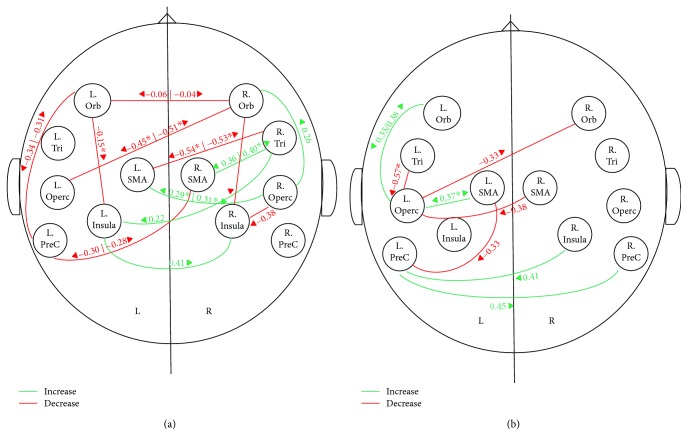
Changes in resting-state connectivity during the treatment interval in the treated patient JV (a) and control patient GB (b). Increase: green; decrease: red. Values represent differences (T3 − T2) in the semipartial correlation. Arrows point to the target region in the calculation of semipartial correlations. Only changes in T3 − T2 which were significantly greater than changes during the baseline period (T2 − T1) are shown. Significance is determined with FDR correction for 66 correlations with *p* < 0.05 or *p* < 0.01 (marked by^∗^). L: left; R: right; Orb: orbitalis; Tri: triangularis; Operc: opercularis; PreC: precentral; SMA: supplementary motor area.

Changes in resting-state connectivity during the treatment interval in the treated patient and the equivalent interval in the control patient are presented in Figure 2 and in the upper half of Tables 4 (for the treated patient) and 5 (for the control patient) in the Supplementary Materials. The values in Figure 2 represent differences in semipartial correlations in T3 − T2. These changes were compared to the changes during the baseline interval (T2 − T1), which are presented in the lower halves of Tables 4 and 5 (in the Supplementary Materials). Only changes that are significantly different between the treatment and baseline intervals are presented in Figure 2. Significance is determined with FDR correction for 66 comparisons (*p* < 0.05 and ^∗^ *p* < 0.01). The results for the treated patient show an increase in the connectivity during the treatment period between regions involved in speech motor control (bilateral SMA and insula) and right frontal language areas. These connections include R.Operc–L.SMA, R.Tri–R.SMA, and R.Tri–L.Insula. There was also an increase in connectivity within the right frontal language areas (R.Orb–R.Operc). These increases were significantly larger than the changes that occurred during the baseline period. Importantly, no increase in connectivity was found for the left language area. In addition to these increases in connectivity, there were also connections showing a decrease in connectivity during the treatment period, and these were for both the left and right language areas (see Figure 2(a)). The pattern of results is altogether different in the control patient, who did not undergo treatment. In this patient, there was no increase in connectivity in the right frontal language areas. Instead, increase in connectivity for the control patient was found between the left frontal language areas and regions involved in speech motor control (L.SMA–L.Operc) and within the left frontal language areas (L.Orb–L.Operc). Finally, both patients showed increases in connectivity between bilateral regions involved in speech motor control, that is, R.Insula–L.Insula in the treated patient and L.PreC–R.PreC and L.PreC–R.Insula in the control patient (see Figures 2(a) and 2(b)).

## 4. Discussion

This study examined the effect of melody-based treatment on a chronic patient with moderate to severe aphasia due to extensive left frontotemporal lesions following two temporally proximal brain injuries three years earlier. The patient's performance on treated and untreated phrases was examined before, immediately after, and eight weeks following treatment. Resting-state connectivity was examined at three time points: T1—baseline (25 months postinjury), T2—pretreatment (36 months postinjury), and T3—posttreatment (39 months postinjury). This was compared to a control patient, who did not receive treatment during the same time period. We expected that improvement in language performance following treatment would be associated with an increase in functional connectivity between right frontal homologues of language areas and regions involved in motor speech control.

Behavioral measures of performance on treated phrases showed improvement in the repetition of sentences of varying lengths and complexity. Likewise, JV's ability to answer question probes improved following treatment, with more appropriate and longer answers; these improvements were maintained at the eight-week follow-up, and similar improvements were not seen in the untreated phrases and/or question probes. Performance on the sentence completion task improved numerically during treatment, but improvement was only marginally significant. Moreover, visual inspection of the data indicated similar levels of improvement in both treated and untreated phrases in the sentence completion task, with no difference between treated and untreated phrases posttreatment. These results, together with improved performance on the responsive naming subtest of the BDAE (a task similar to sentence completion), suggest that treatment effects generalized to untreated stimuli in the sentence completion task (step 4). However, the small number of phrases in step 4 may have underpowered the analysis and masked this effect. Alternatively, the marginal treatment effect for treated phrases in the sentence completion task may suggest that relative to the more open-ended nature of the probe questions, the constrained sentence completion task may have proven especially difficult.

Resting-state connectivity measures for the treated patient showed increases in connectivity between right frontal language areas (R.Tri and R.Operc) and regions involved in speech motor control (bilateral SMA and L.Insula) during the treatment period. There were also significant increases in connectivity within the right frontal language areas (R.Orb–R.Operc), compared to the baseline period. Moreover, these changes were specific to the RH and to the treated patient. In contrast, the control patient, who did not receive treatment, showed increases in connectivity between the left frontal language (L.Operc) and speech motor control area (L.SMA) and within the left frontal language areas (L.Orb–L.Operc) during the same time period.

### 4.1. Behavioral Effect of the Melody-Based Treatment

The behavioral improvements observed in the repetition of treated phrases in the current study are consistent with previous research showing the effectiveness of melody-based treatments for patients with nonfluent aphasia [16, 19]. Although patient JV had focal lesions, she had also suffered from TBI. Except for the two patients reported by Baker [26], other patients reported in the literature who have been treated with MIT or modified versions of MIT have had an etiology of stroke. Our findings extend those of Baker [26] in showing that melody-based therapy can be successful in patients with an etiology other than stroke, which may involve different physiological recovery mechanisms [68]. In addition, the patient's comprehension deficit do not fit the optimal profile for melody-based therapy [18]. Nevertheless, the patient's substantial improvement from the treatment is in keeping with other studies [25, 69] which suggest that relatively intact comprehension ability is not a critical inclusion criterion.

In addition to improvement on treated phrases, our findings also show some evidence for generalization of treatment effects to untreated phrases with the sentence completion task (step 4) and a nonsignificant trend for improvement on the untreated question (step 5). These tasks, which are not typically included in melody-based treatment protocols, such as the standard MIT [19], were introduced in the present study, in order to encourage generalization in the later stages of treatment by practicing the generation of novel words during treatment. That these tasks, and especially the response to questions in stage 5, are similar to natural spontaneous speech is worth noticing. The relatively scarce evidence for generalization to untreated phrases [70] or to spontaneous speech [18, 20, 71] in other MIT studies points to the potential benefit of the sentence completion task in enhancing generalization of treatment effects.

### 4.2. The Effect of the Melody-Based Treatment on the Language Network

Analyses of resting-state connectivity, which show an increase in connectivity within the right frontal language areas, and between these areas and regions involved in speech motor control during treatment, are consistent both with our hypothesis and with the underlying assumptions of melody-based treatment approaches, namely, that musical abilities typically associated with RH areas [22] are preserved in patients with LH lesions and may therefore be recruited to successfully compensate for damaged LH language areas. The current results show that indeed the language network in the RH and specifically the right frontal regions are contributing to improvements seen after treatment.

Our findings are consistent with a number of functional imaging studies, showing a greater increase in activation in the right compared to the left frontal cortex following MIT ([17, 18]; total *N* = 4) although lateralization was not consistent across all functional activation studies ([38–41]; total *N* = 12). The results of the current study extend previous functional imaging findings by showing an increase in the right lateralization at the level of the network. While decreases in RH local activation is ambiguous because it may indicate either greater neural efficiency in the RH or less reliance on the RH [42], the results of functional connectivity measures are more clearly interpretable. Even if local activation decreases due to increased neural efficiency, the coupling between these regions and downstream regions is expected to be enhanced.

By using resting-state data, the current results further demonstrate that these treatment effects are not solely task dependent. Treatment-related changes in resting-state connectivity were previously shown with other types of aphasia therapies [54, 55], and the current study extends them to melody-based therapy. Our results are also consistent with brain stimulation studies showing that excitatory stimulation of the right posterior IFG as an adjuvant to MIT improves the effect of aphasia treatment [15, 47]. In relation to these findings, the current study has further shown that treatment effects are specific to the right hemisphere and to a patient that underwent melody-based therapy. Similar RH language network changes were not seen in the control patient, who showed an increase in connectivity only in LH language areas, and the connections between the LH language network and bilateral areas involved in speech motor control. These increases in connectivity in the control patient, who did not undergo any treatment during the relevant period, may be a result of everyday language experiences this patient may have encountered.

In contrast to functional imaging measures, structural-imaging results indicate a more stable change, which is not task dependent. The evidence, for the involvement of the RH in melody-based treatment in the current study, is broadly consistent with findings from DTI studies showing an increase in the number of fibers in the right arcuate fasciculus in patients following MIT [20, 21]. More specific to the location of the effects within the RH, the increase in connections with the right opercularis (R.Operc–L.SMA; R.Operc–R.Orb) in the current study is consistent with volumetric measures showing a correlation between improvement in intonation-based therapy and increases in white matter volume in the right opercularis [44]. The IFG pars opercularis, which roughly occupies BA 44, serves as the intermediate region between language areas involved in retrieval and the precentral gyrus which is part of the speech motor control system involved in articulation [72, 73]. Within the speech motor control system, SMA plays a critical role in controlling the initiation of speech motor commands [74, 75] and the insula is implicated in articulatory coordination and control [76, 77]. The increase in connectivity between language areas and speech motor control regions (R.Operc–L.SMA; R.Tri–R.SMA; R.Tri–L.Insula), and specifically the convergence on the right opercularis (R.Operc–L.SMA; R.Operc–R.Orb), suggests that the treatment changes the interface between language retrieval and speech motor control. These results are also consistent with studies pointing to slow articulation of connected syllables and auditory-motor integration as the critical ingredient in the effect of MIT [23, 25]. An intriguing suggestion based on these findings is that therapy-induced changes that occur at the interface of the language and speech motor control networks may be more readily generalized to untreated stimuli, as compared to higher-level changes (e.g., word-retrieval processes) within the language network, which may be associated with item-specific learning.

A significant limitation of this study is its single-case design, which does not allow for the correlation of changes seen in brain connectivity and language behaviors. Nevertheless, we believe that the comparison to a baseline period, as well as testing of a control patient enabled us to make conclusions about the specificity of the results to the treatment administered. Lastly, although the measurable focal lesions of this patient were in the left hemisphere and secondary to hemorrhages, the probable presence of diffuse axonal injury secondary to traumatic injury does not allow us to rule out the presence of RH damage; indeed, impairments on measures of visuospatial measures are likely attributable to such damage. Arguably, the RH changes and response to treatment may have been more robust in the absence of these putative changes.

## 5. Conclusions

Our results show the benefit of melody-based treatment for a patient with moderate-severe nonfluent aphasia, more than three years following traumatic brain injury with focal lesions in the left frontotemporal areas due to hemorrhages. The patient's ability to repeat sentences and answer question probes improved on treated items, and this treatment effect was maintained eight weeks following treatment. Improvement in sentence completion was marginally significant and similar for treated and untreated phrases, suggesting generalization. The results of the resting-state imaging suggest that the effect of melody-based treatment is right lateralized. This effect was stable and found at the level of the functional network even during rest and not only in localized task-dependent activation. Our results further show that the effects are treatment specific and are not shown in a patient that did not receive therapy during the relevant period. Beyond the lateralization of treatment, our results further show that melody-based treatment affects the interface between language retrieval and motor speech control and articulation. A treatment affecting this level of processing may be a good basis for generalization to untreated stimuli. Further research is needed in a larger sample of patients with discrete LH focal lesions.

## Figures and Tables

**Table 1:** JV's language assessment 36 months postinjury.

Language assessment	Pretreatment		Posttreatment	
	Raw score	Percentile	Raw score	Percentile
*Boston Naming Test (BNT)—short form*				
BNT—number of spontaneously given correct responses	2/15	30	0/15	30
BNT—number of correct responses following phonemic cue	4/11	NA	4/13	NA
BNT—number of correct choices	3/9	NA	6/11	NA
*BDAE—short form*				
I.A. simple social responses	6/7	50	6/7	50
Aphasia Severity Rating Scale	1/5	40	2/5	50
II.A. word comprehension	8/16	<10	10/16	10
II.B. commands	8/10	40	8/10	40
II.C. complex ideational material	3/6	30	4/6	50
III.B. automatized sequences	1/4	10	1/4	10
III.B. repetition single words	4/5	60	4/5	60
III.B. repetition sentences	1/2	60	1/2	60
III.C. responsive naming	0/10	10	4/10	30
III.C. naming—screening of special categories	7/12	20	7/12	20
IV.C. oral word reading	3/15	<20	3/15	<20
IV.C. oral sentence reading	0/5	30	0/5	30
IV.C. sentence comprehension	0/3	10	0/3	10
IV.D. reading comprehension—sentences and paragraphs	2/4	40	1/4	10
*PPTT (3 pictures)*	40/52	NA	DNT	
*PALPA 7 syllable length repetition*	22/24	NA	22/24	NA

BDAE = Boston Diagnostic Aphasia Examination [57]; PPTT = Pyramids and Palm Trees Test [58]; PALPA = psycholinguistic assessments of language processing in aphasia [59]; NA = percentiles not available.

**Table 2:** JV neuropsychological assessment 25 months postinjury.

Domain/test	Raw score	Standard score	Classification
*Manual motor/psychomotor functioning*			
Grip strength (dom)	25 kg	*T* = 41	Low average
Grip strength (non-dom)	22.5 kg	*T* = 45	Average
Grooved pegboard (dom)	119 sec (0 drops)	*T* = 19	Severely impaired
Grooved pegboard (non-dom)	80 sec (0 drops)	*T* = 42	Low average
*Attention/speed of processing*			
Visual span forwards	8	SS = 9	Average
Symbol Digit Modalities Test—W	42 items (0 errors)	*z* = −1.2	Mildly impaired
Trail Making Test A	40 sec	*T* = 38	Mildly impaired
Trail Making Test B	179 sec (2 errors)	*T* = 23	Moderately impaired
*Language functions*			
MAE Token Test	9/44		Very defective
Peabody Picture Vocabulary Test-III	126	1st %ile	Impaired (age equivalent = 9 years, 9 months)
*Visuospatial functioning*			
Visual Form Discrimination	32/32		Intact
WAIS—Block Design	24/68	SS = 7	Borderline impaired
RVDLT—copy	32/36	6–10%ile	Mildly impaired
RVDLT—time to complete (copy)	361 sec	2-5th %ile	Mildly impaired
RVDLT—copy organizational quality	1/5		Extremely piecemeal; drawn rotated 90 degrees
*Memory*			
RVDLT—total trials 1–5	46 Figures (26 intrusions)	*z* = 0.13	Average
RVDLT—immediate recall	14.5/36	*T* = 37	Mildly impaired
RVDLT—highest number of figures recalled	12/15		
RVDLT—delayed recall	11/15 (4 intrusions)		
RVDLT—delayed recognition	10/15 (2 false alarms)	*z* = −4.7	Severely impaired
BVMT Total Immediate Recall	17/36	*T* = 36	Mildly impaired
BVMT Total—Delayed Recall	8/12	*T* = 44	Average
BVMT Recognition	6/6 (0 false alarms)	>16th %ile	Intact
*Executive functioning*			
Visual span backwards	7	SS = 10	Average
WAIS—matrix reasoning	11/26	SS = 9	Average
WCST—total administered	128 (full WCST)		
WCST—errors	40	*T* = 33	Mildly impaired
WCST—perseverative responses	27	*T* = 31	Mildly impaired
WCST—perseverative errors	23	*T* = 31	Mildly impaired
WCST—nonperseverative errors	17	*T* = 37	Mildly impaired
WCST—conceptual level responses	45	*T* = 31	Mildly impaired
WCST—categories	4	11-16th %ile	Mildly impaired
Trials to complete 1st category	12	>16th%ile	Intact

MAE Token Test = Multilingual Aphasia Examination Token Test; WAIS = Wechsler Adult Intelligence Scale; RVDLT = Rey Visual Design Learning Test; BVMT = Brief Visuospatial Memory Test; WCST = Wisconsin Card Sorting Test; W = written.

**(a):** 

*Sentence repetition (steps 1 to 3)*				
	Mean % correctly produced syllables (SD)			
	Treated phrases (*N* = 21)		Untreated phrases (*N* = 21)	

Pre-	71.34 (28.79)		64.60 (32.59)	
Post-	96.78 (8.05)		61.55 (33.15)	
8-week	93.33 (13.30)		74.11 (30.88)	

*p values (Wilcoxon signed-rank test for related samples)*				
	Treated post	Treated 8 weeks	Untreated post	

Treated pre	*p* = 0.002^∗^	*p* = 0.011^∗^		
Treated post			*p* = 0.001^∗^	

^∗^Alpha = 0.0167 (Bonferroni correction).

**(b):** 

*Sentence completion (step 4)*				
	Mean number of syllables produced (SD)			
	Treated phrases (*N* = 9)		Untreated phrases (*N* = 9)	

Pre-	1.44 (1.88)		1.78 (1.20)	
Post-	4.22 (2.44)		4.78 (2.28)	
8-week	2.78 (2.04)		3.78 (1.72)	

*p values (Wilcoxon signed-rank test for related samples)*				
	Treated post	Treated 8 weeks	Untreated post	Untreated 8 weeks

Treated pre	*p* = 0.02^∧^	*p* = 0.114		
Treated post			*p* = 0.481	
Untreated pre			*p* = 0.048	*p* = 0.026^∧^

^∧^Approaching significance of Alpha = 0.01 (Bonferroni correction).

**(c):** 

*Answering questions (step 5)*				
	Mean number of syllables produced (SD)			
	Treated phrases (*N* = 10)		Untreated phrases (*N* = 10)	

Pre-	0.50 (1.08)		0.40 (0.97)	
Post-	6.20 (2.90)		2.10 (3.03)	
8-week	4.60 (3.06)		2.40 (3.13)	

*p values (Wilcoxon signed-rank test for related samples)*				
	Treated post	Treated 8 weeks	Untreated post	Untreated 8 weeks

Treated pre	*p* = 0.005^∗^	*p* = 0.007^∗^		
Treated post			*p* = 0.010^∗^	
Untreated pre			*p* = 0.068	*p* = 0.066

^∗^Alpha = 0.01 (Bonferroni correction).

Performance on treated and untreated phrases at pre-, post- and 8-week follow-up tests. Sentence repetition (steps 1–3; (a)), sentence completion (step 4, (b)), and probe questions (step 5, (c)) are presented. Statistical comparisons with Wilcoxon signed-rank test are indicated at the bottom of each panel.

## Supplementary Materials

Supplementary Materials Tables 4 and 5 in the supplementary material show changes in resting-state connectivity in the treated and control patients separately for the treatment and baseline periods. These were used to compute the differences in Figure 2. Table 4: changes in resting-state connectivity in the treated patient (JV) during the treatment and baseline periods. Values represent difference in semipartial correlations during the treatment period (T3 − T2 in the upper half) and baseline period (T2 − T1 in the lower half). Rows represent source regions and columns represent target regions in the calculation of semipartial correlations. Values in boldface indicate significant differences between the treatment and baseline periods after FDR correction for 66 comparisons *p* < 0.05 (or *p* < 0.01 in larger font size). L: left; R: right; PreC: precentral; Orb: orbitalis; Tri: triangularis; Operc: opercularis; SMA: supplementary motor area. Table 5: changes in resting-state connectivity in the control patient (GB) during the equivalent of the treatment period and the baseline period. Values represent the difference in semipartial correlations during the equivalent of the treatment period (T3 − T2 in the upper half) and the baseline period (T2 − T1 in the lower half). Rows represent source regions, and columns represent target regions in the calculation of semipartial correlations. Values in boldface indicate significant differences between the equivalent of the treatment period and the baseline periods after FDR correction for 66 comparisons *p* < 0.05 (or *p* < 0.01 in larger font size). L: left; R: right; PreC: precentral; Orb: orbitalis; Tri: triangularis; Operc: opercularis; SMA: supplementary motor area.
