# Supplementary material for: Changes in Resting-State Connectivity following Melody-Based Therapy in a Patient with Aphasia
Source: Neural Plast. 2018 Mar 29;2018:6214095. doi: 10.1155/2018/6214095 (PMC5896238; doi:10.1155/2018/6214095)
Supplement: Supplementary Materials — Tables 4 and 5 in the supplementary material show changes in resting-state connectivity in the treated and control patients separately for the treatment and baseline periods. These were used to compute the differences in Figure 2. Table 4: changes in resting-state connectivity in the treated patient (JV) during the treatment and baseline periods. Values represent difference in semipartial correlations during the treatment period (T3 − T2 in the upper half) and baseline period (T2 − T1 in the lower half). Rows represent source regions and columns represent target regions in the calculation of semipartial correlations. Values in boldface indicate significant differences between the treatment and baseline periods after FDR correction for 66 comparisons p < 0.05 (or p < 0.01 in larger font size). L: left; R: right; PreC: precentral; Orb: orbitalis; Tri: triangularis; Operc: opercularis; SMA: supplementary motor area. Table 5: changes in resting-state connectivity in the control patient (GB) during the equivalent of the treatment period and the baseline period. Values represent the difference in semipartial correlations during the equivalent of the treatment period (T3 − T2 in the upper half) and the baseline period (T2 − T1 in the lower half). Rows represent source regions, and columns represent target regions in the calculation of semipartial correlations. Values in boldface indicate significant differences between the equivalent of the treatment period and the baseline periods after FDR correction for 66 comparisons p < 0.05 (or p < 0.01 in larger font size). L: left; R: right; PreC: precentral; Orb: orbitalis; Tri: triangularis; Operc: opercularis; SMA: supplementary motor area. [file 6214095.f1.docx]

**Supplementary material**

**Table 4.** **Changes in resting state connectivity in the treated patient (JV) during the treatment and baseline periods.** Values represent difference in semi-partial correlations during the treatment period (T3-T2 in the upper half) and baseline period (T2-T1 in the lower half). Rows represent source regions and columns represent target regions in the calculation of semi-partial correlations. Values in boldface indicate significant differences between the treatment and baseline periods after FDR correction for 66 comparisons p<0.05 (or p<0.01 in larger font size). L-Left; R-Right; PreC-Precentral; Orb-Orbitalis; Tri-Triangularis; Operc-Opercularis; SMA-Supplementary motor area.

**Table 5.** **Changes in resting state connectivity in the control patient (GB) during the equivalent of the treatment period and the baseline period.** Values represent difference in semi-partial correlations during the equivalent of the treatment period (T3-T2 in the upper half) and the baseline period (T2-T1 in the lower half). Rows represent source regions and columns represent target regions in the calculation of semi-partial correlations. Values in boldface indicate significant differences between the equivalent of the treatment period and the baseline periods after FDR correction for 66 comparisons p<0.05 (or p<0.01 in larger font size). L-Left; R-Right; PreC-Precentral; Orb-Orbitalis; Tri-Triangularis; Operc-Opercularis; SMA-Supplementary motor area.
